# A GDSL‐motif esterase/acyltransferase/lipase is responsible for leaf water retention in barley

**DOI:** 10.1002/pld3.25

**Published:** 2017-11-03

**Authors:** Chao Li, Guoxiong Chen, Kohei Mishina, Naoki Yamaji, Jian Feng Ma, Fumiko Yukuhiro, Akemi Tagiri, Cheng Liu, Mohammad Pourkheirandish, Nadia Anwar, Masaru Ohta, Pengshan Zhao, Udda Lundqvist, Xinrong Li, Takao Komatsuda

**Affiliations:** ^1^ National Institute of Agrobiological Sciences Tsukuba Ibaraki Japan; ^2^ Shanghai Key Laboratory of Plant Functional Genomics and Resources Shanghai Chenshan Botanical Garden Shanghai China; ^3^ Shanghai Chenshan Plant Science Research Center Chinese Academy of Sciences Shanghai China; ^4^ Key Laboratory of Stress Physiology and Ecology in Cold and Arid Regions Gansu Province China; ^5^ Northwest Institute of Eco‐Environment and Resources Chinese Academy of Sciences Lanzhou China; ^6^ Shapotou Desert Research and Experimental Station Northwest Institute of Eco‐Environment and Resources Chinese Academy of Sciences Lanzhou China; ^7^ Institute of Crop Science NARO Kannondai Tsukuba Ibaraki Japan; ^8^ Institute of Plant Science and Resources Okayama University Kurashiki Japan; ^9^ Crop Research Institute Shandong Academy of Agricultural Sciences Ji'nan China; ^10^ Faculty of Agriculture and Environment Plant Breeding Institute The University of Sydney Cobbitty NSW Australia; ^11^ Nordic Genetic Resource Center (NordGen) Alnarp Sweden

**Keywords:** abiotic stress, cell walls, cuticle/waxes, drought/water stress

## Abstract

The hydrophobic cuticle covers the surface of the most aerial organs of land plants. The barley mutant *eceriferum‐zv* (*cer‐zv*), which is hypersensitive to drought, is unable to accumulate a sufficient quantity of cutin in its leaf cuticle. The mutated locus has been mapped to a 0.02 cM segment in the pericentromeric region of chromosome 4H. As a map‐based cloning approach to isolate the gene was therefore considered unlikely to be feasible, a comparison was instead made between the transcriptomes of the mutant and the wild type. In conjunction with extant genomic information, on the basis of predicted functionality, only two genes were considered likely to encode a product associated with cutin formation. When eight independent *cer‐zv* mutant alleles were resequenced with respect to the two candidate genes, it was confirmed that the gene underlying the mutation in each allele encodes a Gly‐Asp‐Ser‐Leu (GDSL)‐motif esterase/acyltransferase/lipase. The gene was transcribed in the epidermis, and its product was exclusively deposited in cell wall at the boundary of the cuticle in the leaf elongation zone, coinciding with the major site of cutin deposition. CER‐ZV is speculated to function in the deposition of cutin polymer. Its homologs were found in green algae, moss, and euphyllophytes, indicating that it is highly conserved in plant kingdom.

## Abstract 2

Via genetic mapping and comparative transcriptomics, we identified a GDSL‐motif esterase/acyltransferase/lipase gene that was associated with cutin formation and leaf water retention in barley.

## 1 INTRODUCTION

The hydrophobic cuticle covers the surface of the nonwoody aerial organs of land plants. Its function is to limit transpirative water loss; act as a line of defense against UV radiation, dust deposition, pathogen, and insect attack; and also influence plant growth and development, such as preventing organ fusion and male sterility (Yeats & Rose, 2013; Fich, Segerson, & Rose, 2016; Xu et al., 2017). The plant cuticle is typically composed of two hydrophobic components, waxes and cutin (Fernández, Guzmán‐Delgado, Graça, Santos, & Gil, 2016). The cutin polymer deposited on the outside of the polysaccharide cell wall contributes a major structural component of the cuticle, and it is an insoluble linear, dendritic, and/or cross‐linked macromolecule of hydroxy and hydroxy‐epoxy 16‐ and 18‐carbon fatty acids esterified with glycerol, and the classes of the monomers are generally conserved across land plant lineages (Fernández et al., 2016; Fich et al., 2016). The cuticular wax, which is both embedded within and covers the cutin polymer matrix, is composed of very long‐chain fatty acids and their derivatives (Fich et al., 2016; Yeats & Rose, 2013). The association between defective cutin and increased water permeability has been established repeatedly by comparing the performance of mutants and wild types, resulting in the identification of proteins involved in the phenotype: Glycerol‐3‐phosphate acyltransferase 4 and 8 of *A. thaliana* participate in cutin monomer (2‐monoacylglycerol) synthesis (Li et al., 2007; Yang et al., 2012). An ATP‐binding cassette subfamily G transporter HvABCG31/Eibi1 involved in extracellular export of all main cutin monomers in barley (Chen et al., 2011). SHINE transcription factors of both *A. thaliana* and other species (Aharoni et al., 2004; Wang et al., 2012) involved in cutin and wax formation. Tomato *cutin‐deficient 1* (*cd1*) with ∼ 95% reduction in wild‐type fruit cutin load showed drastically increased levels of fruit desiccation (Isaacson et al., 2009). This tomato gene has recently been widely studied by different groups and thus been given different names, such as *CD1* (Yeats et al., 2012), *GDSL1* (Girard et al., 2012), and *CUTIN SYNTHASE 1* (*CUS1*) (Philippe et al., 2016; Yeats et al., 2014). The characterization of CD1 reveals a mechanism of a Gly‐Asp‐Ser‐Leu (GDSL)‐motif esterase/acyltransferase/lipase for the construction of a cutin macromolecule, and the evidence of additional cutin synthases is expected for the presence of appreciable levels of polymeric cutin in the null mutant *cd1* (Yeats et al., 2012).

The GDSL esterases/acyltransferases/lipases contain an N terminal GDS(L) motif, rather than the canonical GXSXG motif characteristic of lipolytic enzymes (Akoh, Lee, Liaw, Huang, & Shaw, 2004). The overall extent of sequence identity between various GDSL enzymes is low, but they harbor five conserved blocks (I–V) which have been used for classification purposes (Upton & Buckley, 1995). GDSL enzymes are well represented in the plant kingdom, with 108 members known in *A. thaliana*, 144 in rice, and 90 in the lycophyte *Selaginella moellendorffii* (Chepyshko, Lai, Huang, Liu, & Shaw, 2012); however, few have yet been characterized in any detail. They participate in a range of processes, including the hydration of pollen on the stigma (Updegraff, Zhao, & Preuss, 2009), germination (Clauss, Baumert, Nimtz, Milkowski, & Strack, 2008), lipid metabolism (Clauss et al., 2008), and cutin deposition (Girard et al., 2012; Hong, Brown, Segerson, Rose, & Roeder, 2017). They also function in the response to both biotic and abiotic stress (Kim et al., 2014).

Mutagenesis in barley has generated a large set (>1,500) of *cer* mutants, the genes underlying which have been assigned to 79 loci (Lundqvist & Lundqvist, 1988). Very recently, the genes underlying *cer‐c*,* cer‐q,* and *cer‐u* mutants with glossy leaf sheaths and spikes and glaucous leaves are identified, and they encode a polyketide synthase, a cytochrome P450 and a hydrolase/carboxylesterase, which are responsible for β‐diketone biosynthesis (Hen‐Avivi et al., 2016; Schneider et al., 2016). Both the near‐isogenic lines (NILs) constructed in a cv. Bowman (BW) background involving *cer‐zv* and *cer‐ym* produce glossy leaf blades, leaf sheaths, and spikes, while *cer‐yl* produces glossy leaf sheaths and spikes and glaucous leaf blades. The three mutants have grains poorly attached to the hulls (Lundqvist, Franckowiak, & Konishi, 1997). Both *cer‐zv* and *cer‐ym* exhibit a poor water retention capacity, attributed to their cutin‐deficient cuticle (Li et al., 2013, 2015). Compared with wild‐type leaves, the levels of four main leaf cutin monomers (ωOH‐9, 10‐epoxy C18, 9(10), 16‐OH C16, ωOH C18:1, and ωOH C16) were reduced by 92%, 82%, 72%, and 61%, respectively, in BW‐NIL (*cer‐zv.268*) and 91%, 78%, 70%, and 63%, respectively, in BW‐NIL (*cer‐ym.753*); about six minor wax components in BW‐NIL (*cer‐zv.268*) and a primary wax component in BW‐NIL (*cer‐ym.753*) were slightly more abundant (Li et al., 2013, 2015). The genes underlying the *cer‐zv* and *cer‐ym* mutations both map to a pericentromeric region of chromosome 4H (Li et al., 2013, 2015), and *cer‐yl* maps to the similar region (Druka et al., 2011). Here, we show that the three mutants proved to be allelic, and an RNA‐Seq approach was taken to isolate the *cer‐zv* gene encoding an GDSL‐motif esterase/acyltransferase/lipase, as this sidesteps the problem of the very large ratio between physical and genetic distance characteristic of the pericentromeric region.

## 2 MATERIALS AND METHODS

### 2.1 Plant materials

Grain of the near‐isogenic lines (NILs), constructed in a BW background for each of the *cer‐ym.753*,* cer‐yl.187,* and *cer‐zv.268* mutants, was obtained from the USDA‐ARS NSGC (Aberdeen, ID, USA). Grain of the primary *cer‐zv.268*,* cer*‐*zv.342,* and *cer*‐*yl.407* mutants, along with their progenitor WT cv. Foma, and of *cer‐ym.130*,* cer‐ym.753*,* cer‐yl.187*,* cer‐yl*.1*88,* and *cer‐yl.821*, along with their progenitor WT cv. Bonus was obtained from NordGen (Alnarp, Sweden). Grain of the wild barley *Hordeum vulgare* subsp. *spontaneum* accession OUH602 was obtained from the Okayama University Institute of Plant Science and Resources (Kurashiki, Japan). F_3_ individuals derived from a set of 20 F_2_ progeny bred from the cross BW‐NIL *cer‐zv.268* × OUH602, which were heterozygous for the gene underlying the mutant phenotype, were used to fine map the gene underlying *cer‐zv,* while F_2_ offspring of the cross OUH602 × BW‐NIL *cer‐ym.753* were used to map the gene underlying *cer‐ym*. Chromosomal locations were validated by reference to the addition lines of barley (cv. Betzes) chromosome (arms) 2H‐7H, 4HS, and 4HL constructed in wheat cv. Chinese Spring (Islam & Shepherd, 1981); the necessary grains were kindly provided by Dr. A.K.M.R. Islam, University of Adelaide, Australia.

### 2.2 Leaf water loss and toluidine blue staining

For a conventional leaf‐drying assay, segments of a fully expanded leaf (~2 cm in length) taken from seedlings at the one‐leaf stage were incubated at the room temperature and photographed after 3 hr. For the time course of leaf‐drying, segments of fully expanded leaves (~8 cm in length) taken from the second leaf of seedlings at the two‐leaf stage were laid on dry paper and held at room temperature for 3 hr; their weight was recorded every 20 min. The loss of water from leaves was expressed as a proportion of the original fresh weight. Each line was represented by five replicates. To determine the permeability of the leaf to the dye toluidine blue, fully expanded second leaves of seedlings at three‐leaf stage were cut and detached segments were immersed in a 0.05% (w/v) solution for 3 hr, and then photographed.

### 2.3 Transmission Electron Microscopy (TEM)

Fully expanded leaves were removed from seedlings at the three‐leaf stage, fixed by immersion in 2.5% (v/v) glutaraldehyde in 0.1 M cacodylate buffer at 4°C for 2 hr, rinsed with the same buffer, after which the solution was replaced by a 2% (w/v) aqueous solution of OsO_4_ and the material held at 4°C for 1 hr. The material was then dehydrated by passage through an ethanol series, transferred to propylene oxide, and embedded in Quetol 812 resin (Nisshin EM, Tokyo). The samples were processed into ultrathin sections, stained with a 4% (w/v) hafnium chloride in methanol, and Sato's lead solution (Sato, 1968). The cutin polymer was visualized using a JEM 1010 TEM device (JEOL, Tokyo, Japan).

### 2.4 Allelism test

The BW‐NILs *cer‐ym.753*,* cer‐yl.187,* and *cer‐zv.268* were intercrossed in a glasshouse because of their low fertility in the field and the resulting F_1_ hybrids (three independent seedlings per cross) were subjected to both the detached water loss test and toluidine blue staining.

### 2.5 *De Novo* markers

New PCR‐based markers targeted to the critical region of the barley genome were developed from the sequences of relevant cv. Morex bacterial artificial chromosomes known to be located in the pericentromeric region on chromosome 4H (www.harvest-blast.org/), from sequences known to map to the relevant region on the basis of the barley Genome (Mayer et al., 2011), and from 14 EST markers presented in the pericentromeric region of the barley chromosome 4H cytological map (Sakata, Nasuda, & Endo, 2010). The chromosomal location of all newly developed markers was confirmed by testing the Chinese Spring‐Betzes chromosome addition line series, along with a sample of 30 F_2_ progeny of the cross BW‐NIL *cer‐zv.268* × OUH602, each of which exhibited the mutant phenotype.

### 2.6 High‐resolution mapping of the genes underlying the *cer‐zv* and *cer‐ym* mutations

Genomic DNA was extracted from young leaves of each mapping population seedling following the method reported by Komatsuda et al. (Komatsuda, Nakamura, Takaiwa, & Oka, 1998). The DNA was typed for both the flanking (AK358684 and AK364819) and the internal (AK370363, AK248269, AK364461, and AK251484) markers identified following prior coarse mapping of the gene underlying the *cer‐ym* mutation (Li et al., 2015), while in the case of the *cer‐zv* locus, the flanking markers were AK370363 and AK364819, and the internal ones were AK248269, AK364461 and AK251484 (Li et al., 2013). Recombinants with respect to these markers were then typed for the *de novo* markers described above (Table S3). The phenotype of each recombinant was identified by applying the leaf water loss assay to a sample of 16–20 progeny obtained by self‐fertilization.

### 2.7 RNA‐Seq analysis and the detection of *Cer‐zv* gene candidates

The template required for RNA‐Seq was extracted, using the TRIzol reagent (Life Technologies, Carlsbad, CA), from partially expanded third leaves of Foma (T1) and *cer‐zv.342* (T2) seedling. The stage was selected, as it corresponds to the peak period of cutin deposition (Richardson et al., 2007). The RNA was submitted for Illumina HiSeqTM 2000 paired‐end sequencing at Biomarker (Beijing, China). Raw reads were subjected to the Illumina 1.3+ quality test, applying a threshold of 30, and the retained sequences were assembled into unigenes using Trinity software version 20130225 (K‐mer = 25). Sequences identified as polymorphic (single nucleotide variants or insertion/deletions) were listed using blastn search (e‐value threshold of 1e‐50, and sequence identity 95%≤). Genome zipper (Mayer et al., 2011; ftp://ftpmips.helmholtz-muenchen.de/plants/barley/integration_hv3h_refGenomesFlcDNAs_sep10.xls) was utilized to pick up candidate genes in *cer‐zv* region. Counting table was produced using linux command (uniq ‐c) from remapped BAM against Foma unigenes and converted into fpkm (fragments per kilobase of transcript per million reads mapped). Functional annotation of candidates was obtained using Blast2Go (release 3.3.5).

### 2.8 Resequencing of *cer* mutants

Eight *cer* mutants, *cer‐zv.342*,* cer‐yl.187*,* cer‐ym.753*,* cer‐ym.130, cer‐yl.188*,* cer‐yl.821*,* cer‐zv.268,* and *cer‐yl.407*, were used for resequencing. RNA samples were extracted from the third leaf EZ of seedlings at the three‐leaf stage using the TRIzol reagent (Life Technologies). The cDNA first strand was synthesized using the SuperScript II system (Invitrogen). RACE PCR (5׳ and 3׳) was used to obtain the full‐length cDNA sequences encoded by cv. Foma, using a GeneRacer kit (Invitrogen), employing the primers detailed in Table S4. The sequencing of gDNA and cDNA templates used gene‐specific primers (Table S5).

### 2.9 Quantitative real‐time PCR (qPCR) assay

The abundance of transcript of the candidates underlying the *cer‐zv* mutation was determined by qPCR, using the Thunderbird SYBR qPCR mix (Toyobo, Osaka, Japan). The relevant primers were designed from the 3′ end of the coding sequences (Table S4). The reference gene was *Actin,* based on primers reported by Sakuma et al. (Sakuma et al., 2013). The assay was conducted on a CFX96 Real‐Time PCR Detection System (Bio‐Rad, Tokyo, Japan), and each assay was represented by three technical replicates of each of four biological replicates. Transcript abundances were calculated from a standard curve, from which relative abundances were obtained, following Sakuma et al. (2013).

### 2.10 RNA in situ hybridization

Amplicons were generated from the coding region (nucleotides 379–697) of the candidate gene T1_Unigene_BMK.48283 sequence from a template of cv. Foma cDNA prepared from the leaf EZ and PrimeSTAR GXL DNA polymerase (Takara, Japan). The relevant primer sequences are given in Table S4. The forward primer included a T3 promoter sequence at its 5′ end to facilitate the synthesis of the antisense strand, while the reverse primer included T7 promoter sequence at its 5′ end to facilitate the synthesis of the sense strand. The subsequent in situ hybridization procedure followed protocols described elsewhere (Komatsuda et al., 2007).

### 2.11 Protein immunostaining

A synthetic peptide NH2‐C+PLNEEVLKKSTSTA‐COOH (positions 123–136 of CER‐ZV) was used to generate polyclonal antibodies in rabbit. The antiserum was passed through a peptide affinity column. Seedlings of cv. Foma and *cer‐zv.342* were grown in 1/5 strength Hoagland's solution for 3 weeks, after which the shoot EZ was sectioned and immunostained as described elsewhere (Yamaji & Ma, 2007). The signal from the secondary antibody (Alexa Fluor 555 goat anti‐rabbit IgG, obtained from Molecular Probes [Eugene, OR, USA]) was captured by confocal laser scanning microscopy.

### 2.12 Phylogenetic analysis

Homologs of CER‐ZV were recovered by a Blastp search (threshold 1e‐10) of Plant GDB (www.plantgdb.org), Sol Genomics Network (http://solgenomics.wur.nl), and IPK Barley BLAST server (http://webblast.ipk-gatersleben.de/barley/viroblast.php), where barley_HighConf_genes_MIPS_23Mar12_ProteinSeq_POPSEQ_BLAST2GO_Mapping.fasta and barley_LowConf_genes_MIPS_23Mar12_ProteinSeq_POPSEQ_BLAST2GO_Mapping.fasta were applied. SignalP 4.1 software (www.cbs.dtu.dk/services/SignalP) was used to identify signal sequences, which were then trimmed. The retained sequences were aligned using ClustalW software, as implemented within the MEGA 6 software package (Tamura, Stecher, Peterson, Filipski, & Kumar, 2013). A maximum‐likelihood tree was constructed employing the partial deletion method and 1,000 bootstrap samples.

## 3 RESULTS

### 3.1 The *cer‐zv* mutant leaf dehydrates readily and forms a defective cuticle

**Figure 1:**
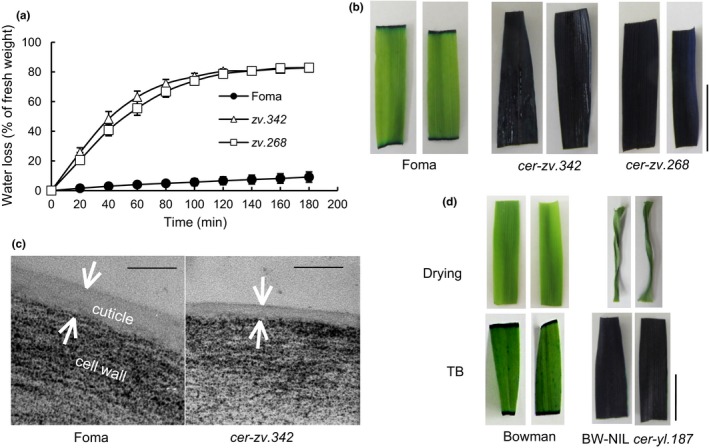
The cuticular phenotype. (a) Water loss assay of detached leaves sampled from *cer‐zv.268, cer‐zv.342,* and cv. Foma. Error bars represent the *SE* (*n* = 5). (b) Toluidine blue staining of leaf segments sampled from *cer‐zv.268, cer‐zv.342,* and cv. Foma. Scale bar: 1 cm. (c) TEM images showing the cuticle in fully expanded seedling leaves of *cer‐zv.342* and cv. Foma, sampled at the three‐leaf stage. Arrows indicate the cuticle thickness. Scale bar: 100 nm. (d) Water loss assay and toluidine blue staining of detached leaves sampled from BW‐NIL *cer‐yl.187* and Bowman. Scale bar: 1 cm

For a conventional leaf‐drying assay, segments of a fully expanded leaf, taken from seedlings at the one‐leaf stage, were incubated at room temperature. Detached leaves sampled from *cer‐zv.268* and *cer‐zv.342* seedlings lost, respectively, 78.7% and 80.4% of their fresh weight after a 2‐hr period of dehydration, compared to a loss of just 6.6% in the wild type (WT) (Figure 1a). The mutants' leaves strongly absorbed toluidine blue, an indication of a highly permeable cuticle, while the WT cuticle was strongly resistant (Figure 1b). As revealed by transmission electron microscopy (TEM), the cuticle formed by the mature *cer‐zv.342* leaf was substantially thinner than that formed by the WT leaf (Figure 1c).

The *cer‐zv* leaves show similar features as *cer‐yl* (Figure 1d) and *cer‐ym* (Li et al., 2013). Detached leaves of BW‐NIL *cer‐yl.187* became severely dehydrated and were highly permeable to toluidine blue (Figure 1d), as do those of both BW‐NIL *cer‐ym*.*753* and BW‐NIL *cer‐zv.268* (Li et al., 2013, 2015).

### 3.2 The *cer‐zv*,* cer‐ym,* and *cer‐yl* Mutants Are Allelic to One Another

**Figure 2:**
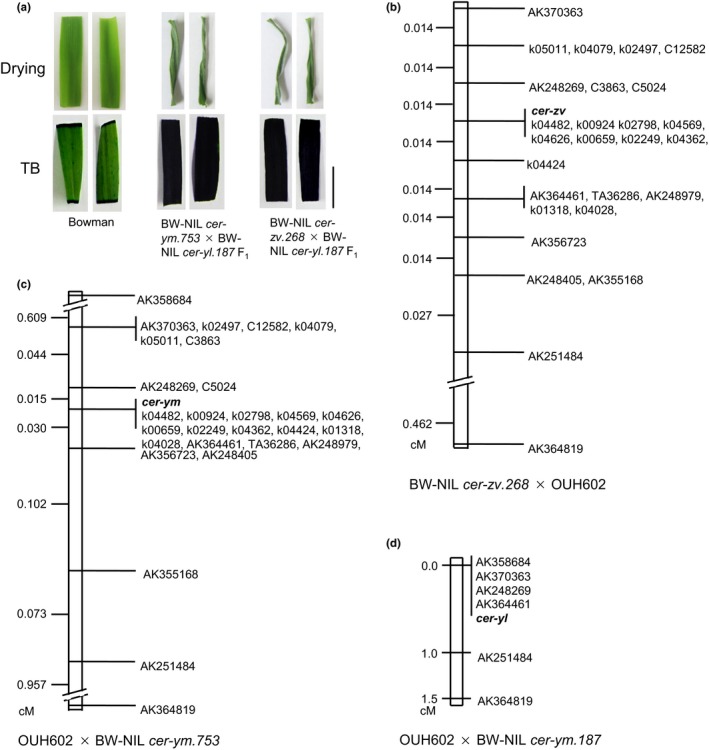
Allelism test and linkage map of *cer‐zv*,* cer‐ym*, and *cer‐yl*. (a) Water loss assay and toluidine blue staining of detached leaves sampled from Bowman and the F_1_ of BW‐NIL *cer‐ym.753* × BW‐NIL *cer‐yl.187* and BW‐NIL *cer‐zv.268* × BW‐NIL *cer‐yl.187*. Scale bar: 1 cm. (b) Fine‐scale mapping of the gene responsible for the *cer‐zv* mutation based on 7,364 gametes sampled from the F_3_ generation of the cross BW‐NIL *cer‐zv.268* × OUH602. (c) Fine‐scale mapping of the gene responsible for the *cer‐ym* mutation based on 6,896 gametes sampled from the F_2_ generation of the cross OUH602 × BW‐NIL *cer‐ym.753*. (d) Coarse‐scale linkage map of the gene responsible for the *cer‐yl* mutation, based on a set of 96 F_2_ progeny bred from the cross OUH602 × BW‐NIL *cer‐yl.187*. The choice of the six markers used was based on their prior localization to the chromosome 4H region harboring the candidate gene (Li et al., 2013)

The lesions induced in *cer‐ym*,* cer‐yl,* and *cer‐zv* have been considered to reflect mutations at three different loci (Lundqvist & Lundqvist, 1988). That the identical gene underlies the three *cer* mutants was established by the F_1_ progeny of the intercrosses, which were conducted in a glasshouse because of their low fertility in the field conditions. All of the F_1_ progeny derived from the cross BW‐NIL *cer‐ym.753* × BW‐NIL *cer‐yl.187* and from BW‐NIL *cer‐zv.268* × BW‐NIL *cer‐yl.187* were unable to prevent massive water loss in the detached leaf test and readily absorbed toluidine blue (Figure 2a). The results indicate that *cer‐zv*,* cer‐ym,* and *cer‐yl* are allelic, and hereafter, *cer‐zv* represents the three mutants.

### 3.3 Fine mapping of *cer‐zv*

To reveal the genetic regions related to the *cer‐zv* and *cer‐ym* mutation, fine mapping was conducted for the two mutants. A fine mapping for *cer‐zv* was carried out using F_3_ progeny bred from twenty F_2_ segregants of the cross BW‐NIL *cer‐zv.268* × OUH602 which were all heterozygous for the mutation. The locus was placed 0.014 cM from AK248269 and 0.014 cM from k04424 based on 7,364 gametes (Figure 2b). Fine mapping of *cer‐ym* was based on OUH602 × BW‐NIL *cer‐ym.753* F_2_ gametes. The gene underlying *cer‐ym* lay 0.015 cM from AK248269 and 0.030 cM from the cosegregating markers AK356723 and AK248405 based on 6,896 gametes (Figure 2c). Molecular markers in *cer‐zv* and *cer‐ym* maps were used for mapping *cer‐yl* with an F_2_ population of OUH602 × BW‐NIL *cer‐yl.187*. The *cer‐yl* was mapped to the pericentromeric region of chromosome 4H which harbors the *cer‐zv* and *cer‐ym* (Figure 2d).

### 3.4 Physical location of *cer‐zv*

The 0.042 cM interval separating k05011 and k04424 (Figure 2b) corresponded to a large proportion (74.4% maximum) of the short arm of chromosome 4H (Fig. S1), indicating a great suppression of recombination in this region. The interval included eight markers (k04482, k00924, k02798, k04569, k04626, k00659, k02249, and k04362) which perfectly cosegregated with the gene underlying *cer‐zv* (Figure 2b) *. *Reference to the barley Genome Zipper (Mayer et al., 2011), some gene‐order inconsistency was noted with the present marker set, implying the possibility of illegitimate recombination or nonhomology‐based translocation event(s). These results indicate that a map‐based cloning approach to isolate the gene was therefore considered unlikely to be feasible.

### 3.5 An RNA‐Seq analysis‐based search for the candidate gene underlying *cer‐zv*

To identify the gene responsible for the mutation in *cer‐zv*, transcriptomes of cv. Foma (T1) and *cer‐zv.342* (T2) were compared using RNA‐Seq. Alignment of the two sets of unigenes revealed 263 unigene pairs with 363 variation sites causing amino acid changes in *cer‐zv.342* (Table S1.1). The overlap between the 263 unigenes with nonsynonymous variation and 583 genes identified from the Genome Zipper chromosome 4H *cer‐zv* region (42.45–48.72 cM) (Mayer et al., 2011), a large region covering the *cer‐zv* map from AK370363 to AK251484 (Figure 2b), reduced the length of the candidate list to five genes (Table S1.2). Two candidate unigenes, T1_Unigene_BMK.48283 and T1_Unigene_BMK.44400, were identified from prior functional annotation as being possibly relevant for cutin deposition: these encoded, respectively, a GDSL‐motif esterase/acyltransferase/lipase and an ABC transporter F family member 5‐like protein. The two candidate genes for *Cer‐zv* were therefore named *HvGDSL1* and *HvABCF1*.

### 3.6 Identification of the gene underlying *cer‐zv* through resequencing

**Figure 3:**
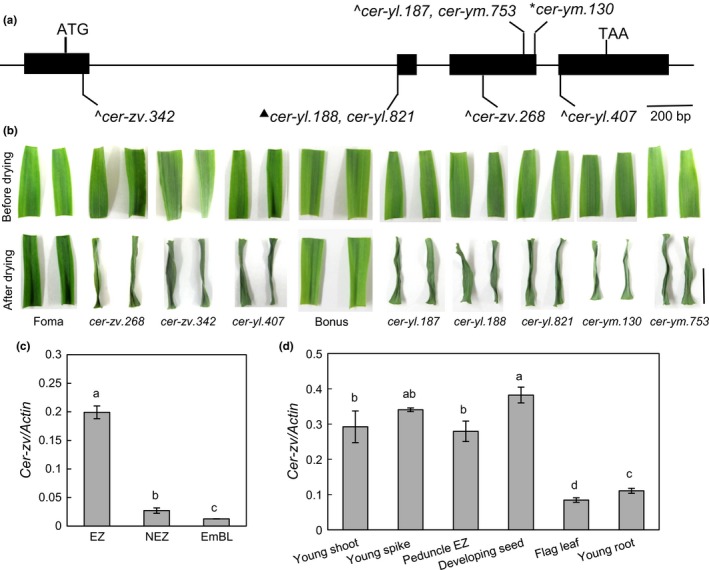
The mutation sites, mutant phenotype, and transcript levels of *HvGDSL1*. (a) Exon/intron structure of the *HvGDSL1* gene, showing the lesions induced in *cer‐zv* mutants. Alterations affecting the open‐reading frame: nonsynonymous single base changes indicated by ^, indels generating a frame shift by *, base substitutions at the splicing site generating a frame shift by^▲^. *cer‐zv.342* caused G25R, *cer‐yl.188,* and *cer‐yl.821* a wrong splicing at A36, *cer‐zv.268* K108M, *cer‐yl.187* D167V, *cer‐ym.753* D167Y, *cer‐ym.130* a wrong splicing at T184, and *cer‐yl.407* D185N. (b) Water loss in detached leaves after 3 hr of WT and *cer* mutants. Scale bar: 1 cm. (c) Transcript levels in the cv. Foma leaf EZ (elongation zone), NEZ (nonelongation zone), and EmBL (emerged blade), prepared from the third leaf of seedlings sampled at the three‐leaf stage. Error bars represent the *SE* (*n* = 4). (d) Transcript levels in the young shoot (including the plumule and coleoptile), the immature spike, the peduncle EZ, the developing seed, the flag leaf prior to anthesis, and the young root. Error bars represent the *SE* (*n* = 3). The same letters above each column indicate the means are not significantly different

Eight independent *cer‐zv* mutants, *cer‐zv.268*,* cer‐zv.342*,* cer‐ym.130, cer‐ym.753*,* cer‐yl.187*,* cer‐yl.188*,* cer‐yl.407,* and *cer‐yl.821*, were used for resequencing of the two candidate genes. Mutations within the *HvGDSL1* open‐reading frame were identified in all eight mutants (Figure 3a, Table S2). All eight mutants were compromised with respect to cuticle permeability in the seedling leaf (Figure 3b). The length of the entire gene was 2780 nt and was split into four exons; its predicted product was a protein with 254 residues, denoted here as CER‐ZV/HvGDSL1 (NCBI accession number BAJ94978). A blastp search highlighted a number of closely related sequences, namely the *A. thaliana* At3 g11210 (62.7% identity), the tomato Solyc06 g051720.2.1 (70.5% identity), and the rice Wilted Dwarf and Lethal 1 (WDL1, 86.5% identity) (Park et al., 2010) (Fig. S2).

With respect to the second candidate *HvABCF1*, the only open‐reading frame sequence variant detected among the eight mutants was in *cer‐zv.342*, which harbored a V98M mutation; this site is located within the N terminal side of the predicted ATP‐binding cassette domain. According to an analysis based on SNAP prediction software (Hecht, Bromberg, & Rost, 2015), the effect of this mutation was neutral (a score of −25 and an expected accuracy of 61%). The implication was that this gene was unlikely to underlie the *cer* mutations.

### 3.7 Spatiotemporal detection of *Cer‐zv* transcript and translation product

**Figure 4:**
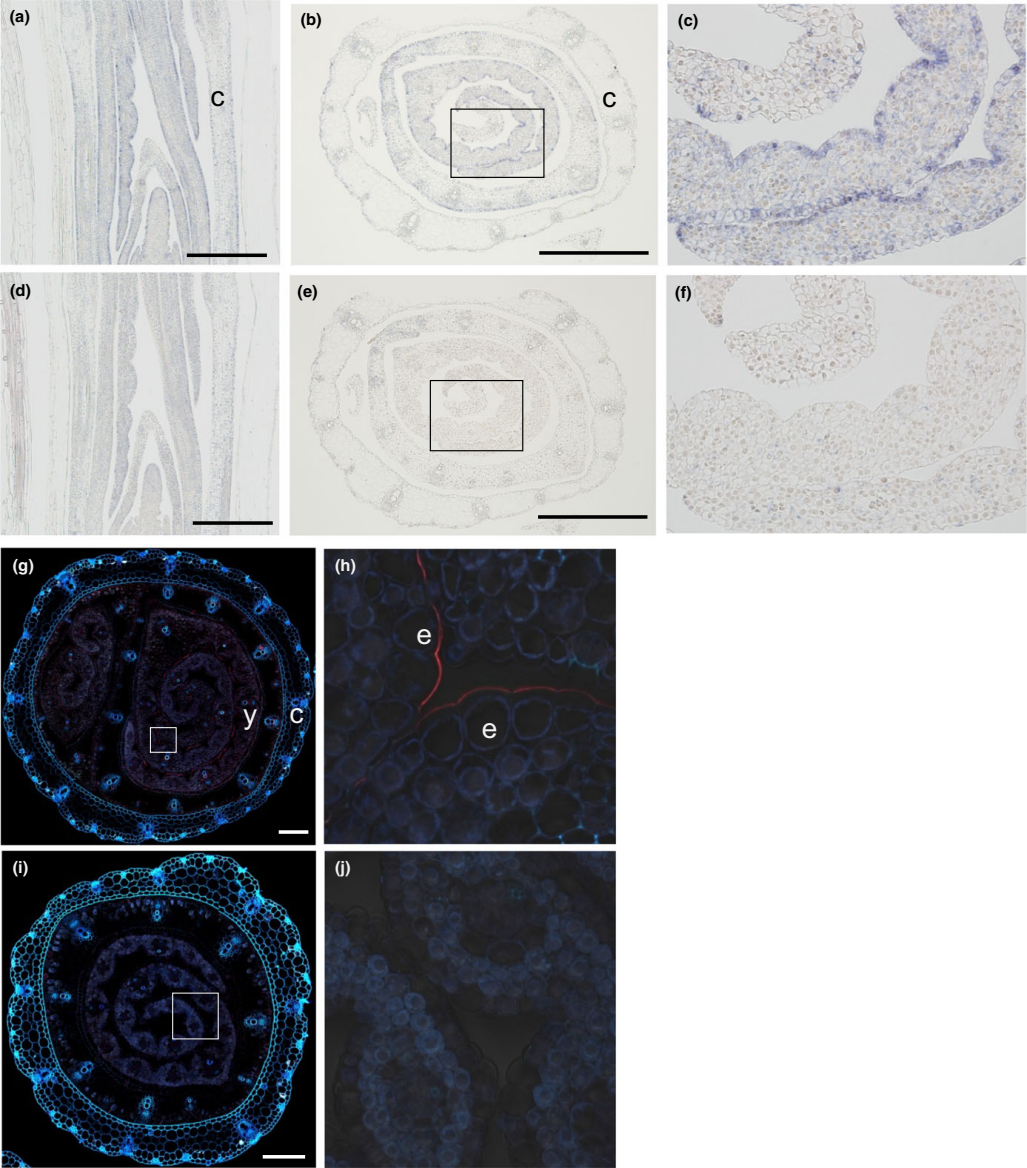
RNA in situ hybridization of the *HvGDSL1* gene and the immunofluorescence‐based localization of its product CER‐ZV. Longitudinal and transverse sections of cv. Foma three‐leaf stage seedlings probed with (a–c) the antisense RNA and (d–f) the sense RNA sequence. The sections shown in (b) and (e) were taken 5–15 mm above the root–shoot junction, and the images (c) and (f) represent an enlargement of the boxed regions in, respectively, (b) and (e). Scale bar: 500 μm. (g–j) The immunofluorescence‐based localization of CER‐ZV in a transverse section taken 5–15 mm above the root–shoot junction of cv. Foma (g, h) and *cer‐zv.342* (i, j) seedlings sampled at three‐leaf stage. The images in (h) and (j) represent an enlargement of the boxed regions in, respectively, (g) and (i). c—coleoptile, e—epidermal cell, y—young leaf. The fluorescence signal appears red. Scale bar: 200 μm

A qPCR analysis revealed that *HvGDSL1* was abundantly transcribed in the third leaf elongation zone (EZ), but the transcript was only present in trace amounts in both the nonelongation zone (NEZ) and the emerged blade (EmBL) (Figure 3c). The gene was also strongly transcribed in the developing seed and young spike, young shoot, and peduncle elongation zone with only trace activity detected in the flag leaf and young root (Figure 3c). An RNA in situ hybridization experiment confirmed that the transcript was abundant in the young leaf tissues of WT seedlings, but not in their coleoptiles (Figure 4a, b, d, e) and that transcript was accumulated in the epidermis (Figure 4c, f), precisely where the cutin forms. Immunostaining with an antibody against this protein indicated that it was exclusively deposited in cell wall at the boundary of the cuticle of the WT epidermis (Figure 4g–j), which imply that the protein is synthesized within the epidermal cells and then secreted to the outer cell walls, a property shared with many GDSL enzymes (Akoh et al., 2004).

### 3.8 Conservation of the CER‐ZV sequence across plant kingdom

A phylogenetic analysis of the homologs of CER‐ZV was conducted (Fig. S3). The homologs harbored by *P. patens*,* S. moellendorffii,* and *V. carteri,* shared, respectively, 50%, 45%, and 30% sequence identity with CER‐ZV, sufficient to suggest that their ancestral gene is ancient and has been retained in many land plants. The CER‐ZV sequence showed high homology with rice WDL1, tomato Solyc06g051720.2.1, and *A. thaliana* At3g11210, about 87%, 71%, and 63%, respectively. The high sequence similarity indicates the potential functional conservation of the enzyme in monocot and dicot plants.

## 4 DISCUSSION

The barley *cer‐zv* and *cer‐ym* mutants are all dwarfed and are compromised with respect to their ability to retain a hydrated state. In each case, the cuticle formed is thinner than the wild type, and its cutin content is low (Li et al., 2013, 2015). Here, evidence has been presented to show that the gene underlying the phenotype of the eight independent *cer‐zv* mutants, *cer‐zv.268*,* cer‐zv.342*,* cer‐ym.130, cer‐ym.753*,* cer‐yl.187*,* cer‐yl.188*,* cer‐yl.407,* and *cer‐yl.821*, encodes a GDSL‐motif esterase/acyltransferase/lipase (HvGDSL1/CER‐ZV) and that the HvGDSL1 is responsible for the retention of leaf water in barley.

Within barley, the gene has no closely related homologs. The only gene in rice which resembles it (87% sequence identity) is *WDL1*; this gene maps to a segment of chromosome 11 which is syntenous with the part of chromosome 4H housing the *Cer‐zv* candidate gene (Mayer et al., 2011). WDL1 is involved in cutin polymer organization in rice (Park et al., 2010). The loss‐of‐function *wdl1* mutant produces a more extreme phenotype than that of *cer‐zv*: The plant is stunted and generally dies during the seedling stage due to its inability to retain water; its cuticle is loosely packed and of irregular thickness. The poorer ability of rice (compared to barley) to tolerate cutin deficiency also applies to the related mutants *eibi1* in barley and *Osabcg31* in rice (Chen et al., 2011). Eibi1 is a HvABCG31 full transporter involved in cutin polymer formation. The *eibi1* mutant also increases water loss from leaves. The *Eibi1* gene is highly expressed, and its product is detected in the EZ of a growing leaf, but not in the NEZ and EmBL (Chen et al., 2011). The cutin deposition occurs in the epidermis of the barley leaf EZ, whereas wax deposition takes place in the NEZ and EmBL (Richardson et al., 2007). Both the transcript and gene products derived from the *cer‐zv* candidate gene were present in the epidermis and more particularly in its outermost cell layer where the cuticle is formed. Transcript and protein were highly abundant in the EZ where much of the plant's cutin is deposited. The HvGDSL1/CER‐ZV protein was exclusively deposited in cell wall at the boundary of the cuticle. A signal peptide is predicted in the protein using Phobius prediction tool (http://www.ebi.ac.uk/Tools/pfa/phobius/). However, according to SignalP4.1 prediction software (http://www.cbs.dtu.dk/services/SignalP/), CER‐ZV does not highlight any peptide signal. Nevertheless, the homologous protein AT3G11210 does not display a signal peptide and has been isolated in Arabidopsis apoplasm (Ge et al., 2011). The cuticle thickness was substantially reduced in *cer‐zv* leaves as revealed by TEM. The cuticle seen in the TEM primarily consists of cutin polymer and/or cutan because of the depletion of extracuticular wax during the conventional chemical fixation (Shumborski, Samuels, & Bird, 2016). Cutan is a highly insoluble and non‐deesterifiable residue of isolated cuticles; it contains mostly of aliphatics linked by ether and/or C–C bonds. It also contains aromatics and cell wall carbohydrates (Pollard, Beisson, Li, & Ohlrogge, 2008). To which degree barley leaf cuticle contains a cutan remains unknown. The reduced cuticle thickness detected in the present study correlates well with a substantial reduction in the amounts of the major cutin monomers in both *cer‐zv* and its allelic mutant *cer‐ym* as demonstrated in our previous studies (Li et al., 2013, 2015). One may infer that the thickness of cutin polymer in *cer‐zv* might be reduced and speculate that HvGDSL1/CER‐ZV may function in cutin polymer deposition.

The transcription profile and the localization of the *Cer‐zv* product both resemble those associated with the tomato fruit cutin synthesis gene *CD1/GDSL1/GDSL2* encoding a GDSL protein (Yeats et al., 2012; Girard et al., 2012; Petit et al., 2014). The *cd1* mutant similarly features a thin cuticle, a reduction in the presence of cutin monomers and increased sensitivity to water loss (Yeats et al., 2012). In *GDSL1*‐silenced tomato lines, a decrease in fruit cuticle thickness and reductions in cutin monomer content and cutin density were exhibited. An increased water permeability is observed in fruit cuticles of the severely silenced transgenic lines (Girard et al., 2012). The *gdsl2‐b* is a strong cutin‐deficient mutant. Alterations of cutin biosynthesis in this mutant are also related to the cuticle permeability to water (Petit et al., 2014). However, CER‐ZV and CD1/GDSL1*/*GDSL2 lack sequence similarity. The fruit cuticle of the *cd1* null mutant still shows certain amounts of polymeric cutin, implying the presence of an additional cutin synthase (Fich et al., 2016; Yeats et al., 2012). TC172499, the tomato homolog of CER‐ZV (sharing 70% sequence identity), is particularly abundant in the peels of developing fruit, a site of high cutin content (Mintz‐Oron et al., 2008). Whether this CER‐ZV homolog functions in the deposition of cutin polymer remains to be determined. Another characterized *GDSL* in plant is the gene encoding cutin synthase 2 (CUS2) in Arabidopsis (Hong et al., 2017). The loss of function of *CUS2* leads to significant reductions of several of the cutin monomers, including ρ‐coumaric acid and α,ω‐dicarboxylic acids, in the inflorescences. The *cus2‐1* flowers (including sepals and petals) exhibit an increase in cuticle permeability as they are stained dark blue when immersed in a toluidine blue solution. It may be concluded that a few *GDSL* genes are involved in cutin polymer formation and cuticle permeability.

GDSLs across the plant kingdom are clustered into two major groups (A and B) and four minor groups (C, D, E, and F) based on the analysis of 533 protein sequences from proteomes of 12 plants, including algae (*V. carteri*), moss (*P. patens*), bryophyte (*S. moellendorffi*), *Arabidopsis thaliana*, rice (*O. sativa*), and other land plants (Vujaklija et al., 2016). Groups A, B, and C contain GDSLs from land plants, whereas groups E and F arise from algal sequences, and only group D includes both land plants and algae. Group D proteins may conserve an essential function, while group A, B, and C proteins possibly gain specialized functions. The two cutin deposition‐related GDSLs, barley CER‐ZV and tomato CD1, belong to groups D and A, respectively, as clarified by group‐specific motifs (Vujaklija et al., 2016). Thus, CER‐ZV is speculated to function essentially and CD1 specifically. In green algae, representing the presumed originator of all terrestrial plants (Wodniok et al., 2011), the species *V. carteri* carries a CER‐ZV homolog protein Vocar20004404m that is identified with an E value of 1e‐35 and an identity of 30%, but no CD1 homolog could be found, which indicate that CER‐ZV is broadly conserved in plant kingdom.

Four distinct single residue changes to the CER‐ZV sequence induced the *cer* phenotype, implying that each of them resides within a functional motif. The D185N substitution (*cer‐yl.407*) involved the replacement of a neutral by an acidic residue and so can be expected to affect block V functionality. The other three substitutions affected residues which were neither any of the known catalytic residues nor lay within blocks I‐III or V. The site of the G25R substitution in *cer‐zv.342* is the fourth residue of a GGWGA motif shared by the homologs. This site is important for the function of CER‐ZV protein because its mutation caused the altered phenotype in the *cer‐zv.342* plants. The same amino acid substitution in the other proteins also affects the function of the corresponding proteins. For example, The G2032R mutation (not gatekeeper residue) identified in crizotinib‐resistant *ROS1* translocations tumors is classified as a strong resistance mutation through steric interference with drug binding (Awad et al., 2013). The K108M substitution in *cer‐zv.268* disrupts the HLKSLS motif present in most homologs. Similarly, the p‐hydroxybenzoate hydroxylase (PHBH) K297M mutation results in decreased catalytic ability particularly in the oxidative half‐reaction (Moran, Entsch, Palfey, & Ballou, 1997). The D167V (*cer‐yl.187*) and D167Y (*cer‐ym.753*) substitutions affect a conserved KVVDLW motif. The assumption here is that the replacement of the acidic D residue by the nonpolar V or Y is responsible for the changed activity of the gene product. In a similar case, the c‐kit receptor tyrosine kinase (KIT) mutant with D814 substituted by V or Y in the phosphotransferase domain is found to generate oncogenic protein with highly constitutively activating phosphorylation and degradation (Moriyama et al., 1996). The data suggest that the three residues, G25, K108, and D167, lying outside the well‐recognized blocks may reside in regions essential for the correct binding of substrates, catalytic ability, or stability of CER‐ZV. Further investigations are needed to elucidate how these mutations affect the activity of CER‐ZV.

## 5 CONCLUSION

*Cer‐zv* encodes a GDSL protein with a speculated function in the deposition of cutin polyester which is associated with the water retention in the barley leaf. Three distinct single sites are found to be vital for the function of CER‐ZV protein. Although it is clear that the loss of function of *Cer‐zv* produces a defective cutin polymer, an important future experiment will be to assay the enzyme activity of CER‐ZV in vitro. The present study reveals the significance of an intact cutin polymer in protecting leaves from nonstomatal water loss and the crucial evolution of CER‐ZV homologs in plant land colonization.

## AUTHOR CONTRIBUTIONS

G.C. and T. K. conceived the original research plans and supervised the experiments; C.L., G.C., N.Y., J.F.M., F.Y., A.T., C.L., M.P., N.A., M.O., P.Z., U.L., and X.L. performed the experiments; C.L., G.C., and K.M. analyzed the data; C.L, G.C., T.K., K.M., and J.F.M. wrote the manuscript.
