# Supplementary material for: A GDSL‐motif esterase/acyltransferase/lipase is responsible for leaf water retention in barley
Source: Plant Direct. 2017 Nov 3;1(5):e00025. doi: 10.1002/pld3.25 (PMC6508521; doi:10.1002/pld3.25)
Supplement: Supplementary file 1 [file PLD3-1-e00025-s001.pptx]

## Slide 1
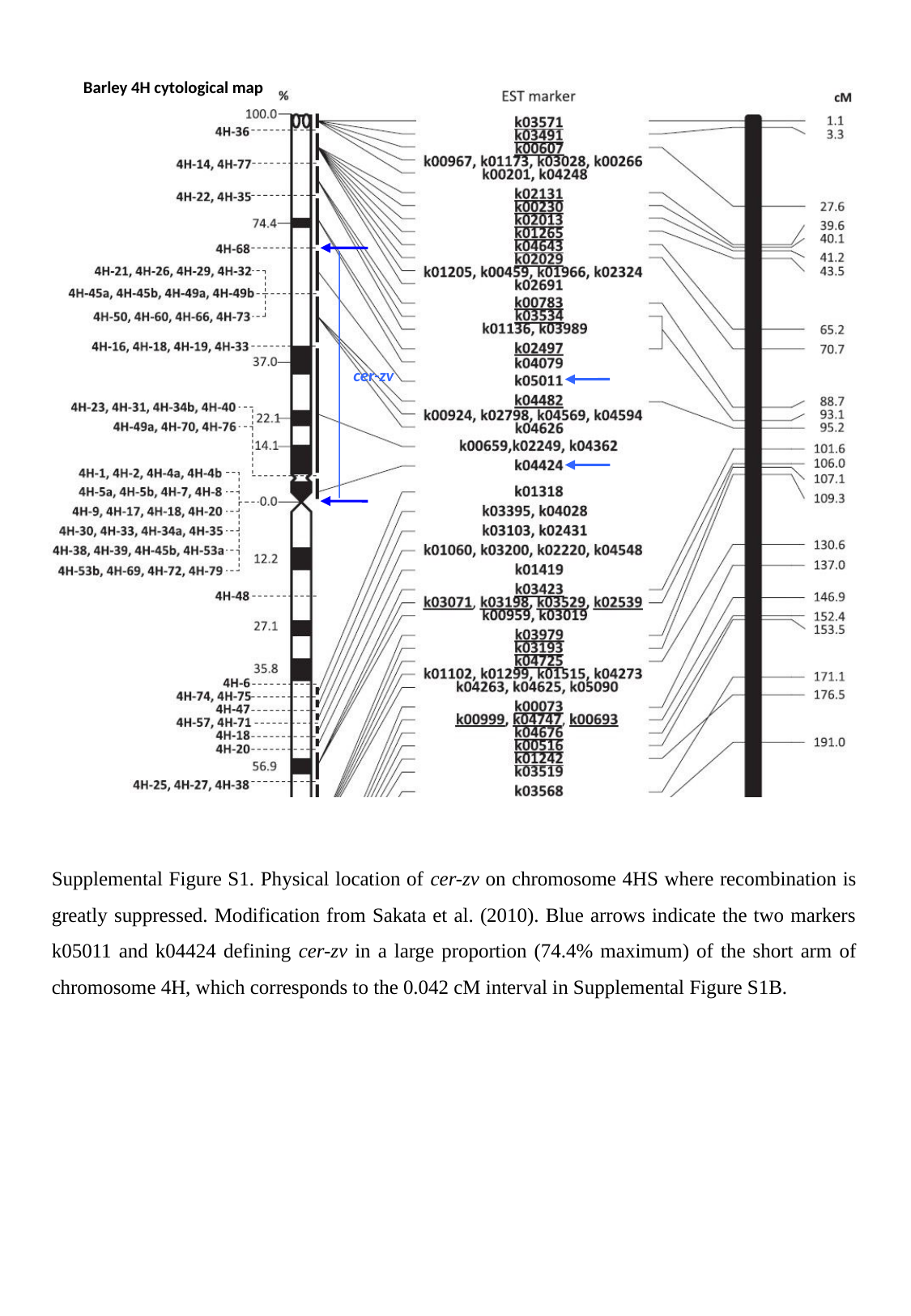

Barley 4H cytological map
cer-zv
Supplemental Figure S1. Physical location of cer-zv on chromosome 4HS where recombination is greatly suppressed. Modification from Sakata et al. (2010). Blue arrows indicate the two markers k05011 and k04424 defining cer-zv in a large proportion (74.4% maximum) of the short arm of chromosome 4H, which corresponds to the 0.042 cM interval in Supplemental Figure S1B.
