# Supplementary material for: A GDSL‐motif esterase/acyltransferase/lipase is responsible for leaf water retention in barley
Source: Plant Direct. 2017 Nov 3;1(5):e00025. doi: 10.1002/pld3.25 (PMC6508521; doi:10.1002/pld3.25)
Supplement: Supplementary file 2 [file PLD3-1-e00025-s002.pptx]

## Slide 1
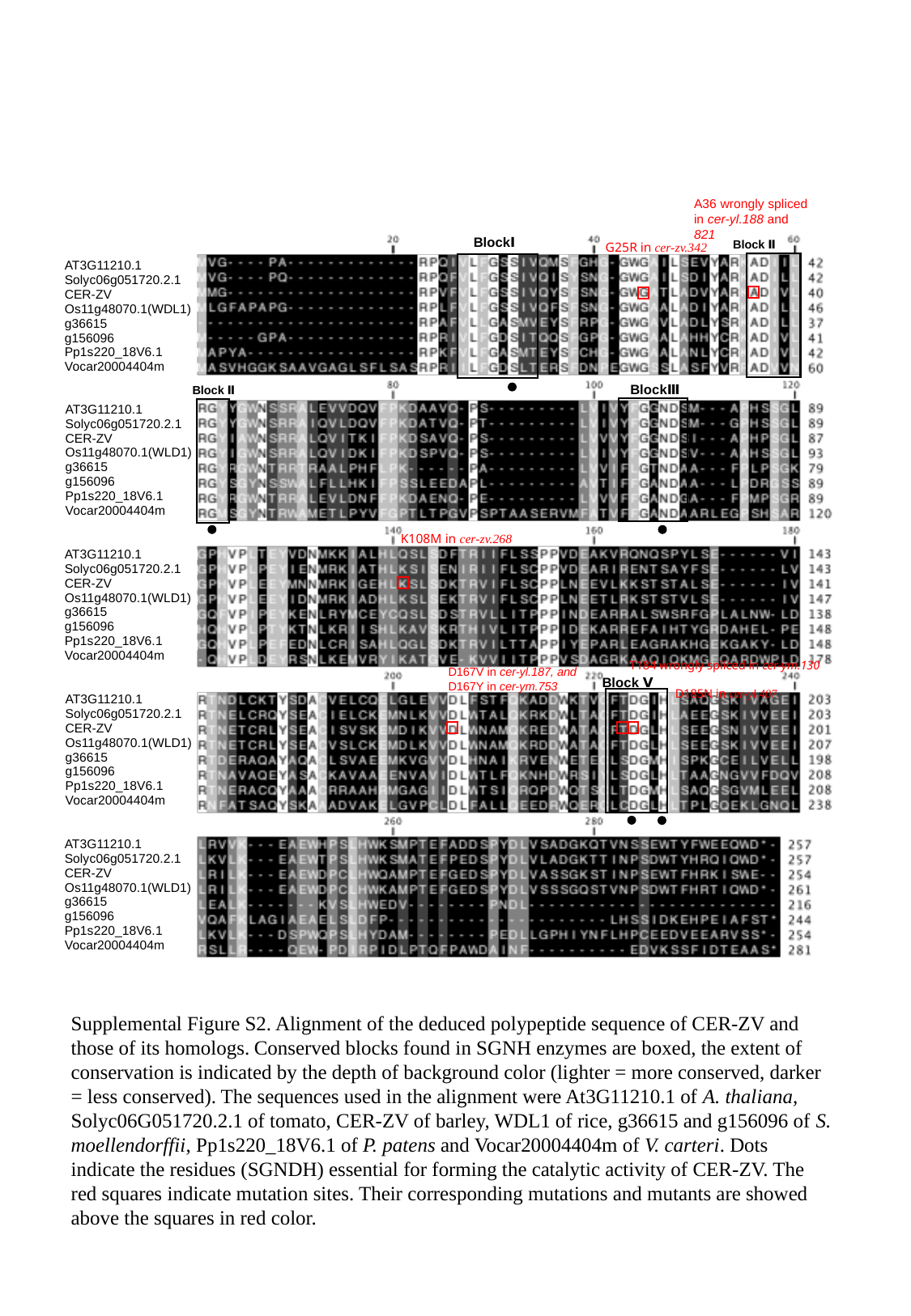

A36 wrongly spliced in cer-yl.188 and 821
BlockⅠ
Block Ⅱ
G25R in cer-zv.342
AT3G11210.1
Solyc06g051720.2.1
CER-ZV
Os11g48070.1(WDL1)
g36615
g156096
Pp1s220_18V6.1
Vocar20004404m
BlockⅢ
Block Ⅱ
AT3G11210.1
Solyc06g051720.2.1
CER-ZV
Os11g48070.1(WLD1)
g36615
g156096
Pp1s220_18V6.1
Vocar20004404m
K108M in cer-zv.268
AT3G11210.1
Solyc06g051720.2.1
CER-ZV
Os11g48070.1(WLD1)
g36615
g156096
Pp1s220_18V6.1
Vocar20004404m
D167V in cer-yl.187, and
D167Y in cer-ym.753
T184 wrongly spliced in cer-ym.130
 D185N in cer-yl.407
Block Ⅴ
AT3G11210.1
Solyc06g051720.2.1
CER-ZV
Os11g48070.1(WLD1)
g36615
g156096
Pp1s220_18V6.1
Vocar20004404m
AT3G11210.1
Solyc06g051720.2.1
CER-ZV
Os11g48070.1(WLD1)
g36615
g156096
Pp1s220_18V6.1
Vocar20004404m
Supplemental Figure S2. Alignment of the deduced polypeptide sequence of CER-ZV and those of its homologs. Conserved blocks found in SGNH enzymes are boxed, the extent of conservation is indicated by the depth of background color (lighter = more conserved, darker = less conserved). The sequences used in the alignment were At3G11210.1 of A. thaliana, Solyc06G051720.2.1 of tomato, CER-ZV of barley, WDL1 of rice, g36615 and g156096 of S. moellendorffii, Pp1s220_18V6.1 of P. patens and Vocar20004404m of V. carteri. Dots indicate the residues (SGNDH) essential for forming the catalytic activity of CER-ZV. The red squares indicate mutation sites. Their corresponding mutations and mutants are showed above the squares in red color.
