# Supplementary material for: A GDSL‐motif esterase/acyltransferase/lipase is responsible for leaf water retention in barley
Source: Plant Direct. 2017 Nov 3;1(5):e00025. doi: 10.1002/pld3.25 (PMC6508521; doi:10.1002/pld3.25)
Supplement: Supplementary file 3 [file PLD3-1-e00025-s003.pptx]

## Slide 1
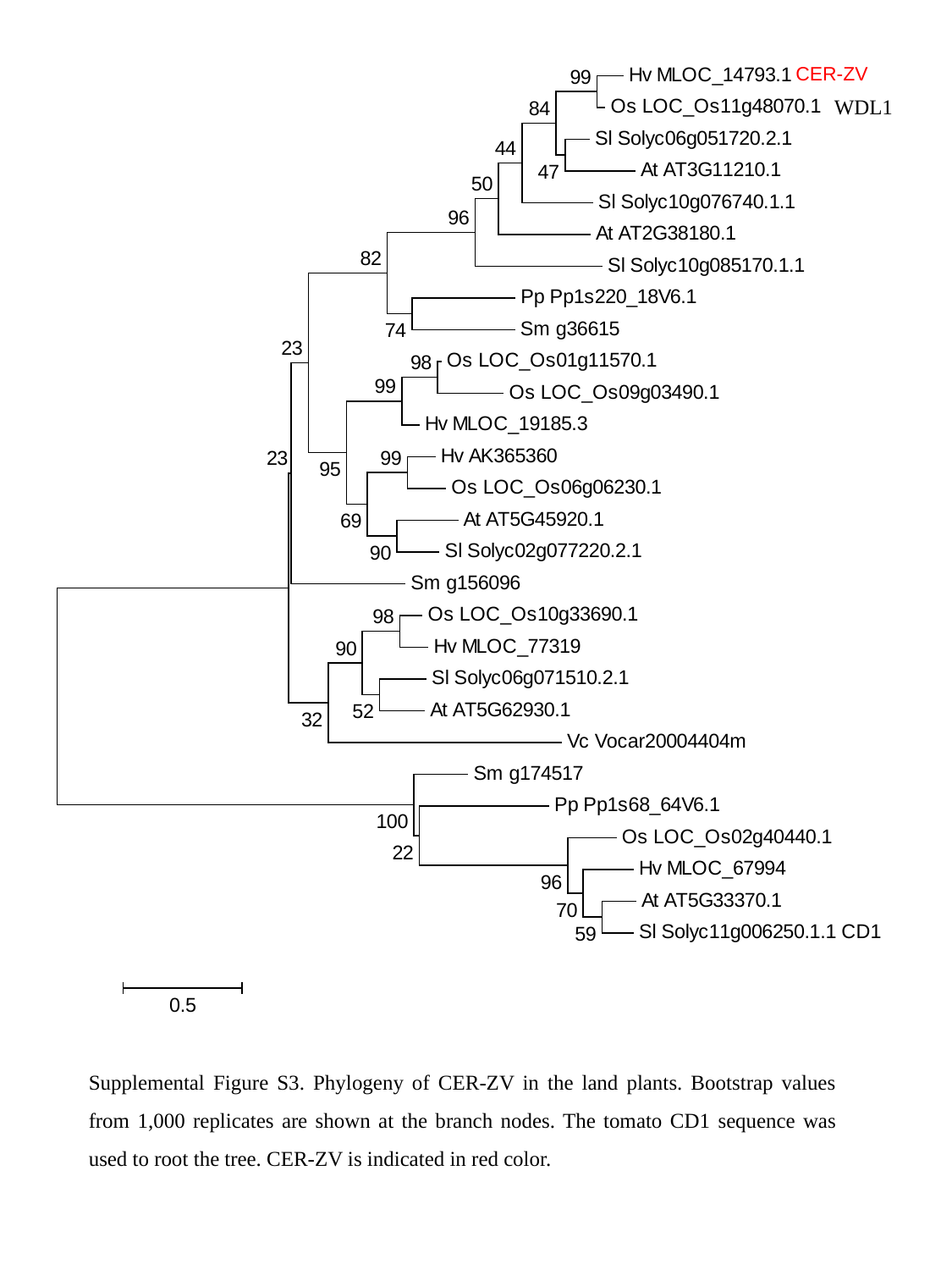

CER-ZV
WDL1
Supplemental Figure S3. Phylogeny of CER-ZV in the land plants. Bootstrap values from 1,000 replicates are shown at the branch nodes. The tomato CD1 sequence was used to root the tree. CER-ZV is indicated in red color.
