# Supplementary material for: A GDSL‐motif esterase/acyltransferase/lipase is responsible for leaf water retention in barley
Source: Plant Direct. 2017 Nov 3;1(5):e00025. doi: 10.1002/pld3.25 (PMC6508521; doi:10.1002/pld3.25)
Supplement: Supplementary file 5 [file PLD3-1-e00025-s005.docx]

Supplemental Table S1. Unigenes showing sequence polymorphism between cv. Foma and *cer-zv.342*. (This Table S1 is an excel file that is submitted in a separate file.)

Supplemental Table S2. Mutational events induced within the *HvGDSL1* open reading frame (candidate for gene underlying the *cer-zv* mutation) sequence.

| Mutant # | Accession | Original cv. | Mutagen | DNA | Protein |
| --- | --- | --- | --- | --- | --- |
| *cer-zv.268* | NGB111155 | Foma | Ethyleneimine | 1972 A > T | K108 > M |
| *cer-zv.342* | NGB111229 | Foma | Ethyleneimine | 247 G > A | G25 > R |
| *cer-ym.130* | NGB111015 | Bonus | Ethylene oxide | 2202-2223 deletion | Splicing site |
| *cer-ym.753* | NGB111641 | Bonus | Ethylene oxide | 2148 C > G and 2149 G > T | Null and D167 > Y |
| *cer-yl.187* | NGB111073 | Bonus | Propane disulfonic acid diethyl ester | 2150 A > T | D167 > V |
| *cer-yl.188* | NGB111074 | Bonus | Propane disulfonic acid diethyl ester | 1606 A >T | Splicing site |
| *cer-yl.407* | NGB111295 | Foma | Propane disulfonic acid diethyl ester | 2299 G > A | D185 > N |
| *cer-yl.821* | NGB111709 | Bonus | Propane disulfonic acid diethyl ester | 1606 A >T | Splicing site |

Supplemental Table S3. Molecular markers used for fine scale mapping.

| Markers | Forward Primer (5’ to 3’) | Reverse primer (5’ to 3’) | Marker type | Restriction enzyme |
| --- | --- | --- | --- | --- |
| AK248979^a^ | CAGTCCAACAACCCAAACCT | AAGGGAACGTGACAAGCATC | Sequencing | – |
| AK356723^a^ | CGGACAAGGAGAAGGAGAAAG | CTAGCGACCGAAACACACG | CAPs | *HhaI* |
| AK248405^a^ | CAATAAAGGGCGTGTGCTG | TCACCTCCAAAAGGACGAA | CAPs | *SspI* |
| AK355168^a^ | CGGAAGGAACTAATATGGATCG | CAAGCAAACCACAGCCACT | SSR | *–* |
| C12582^b^ | CATGCGACAAAAATGTGACC | TAGGCTGGCTCATCGAAACT | Sequencing | *–* |
| C3863^b^ | GAGGTGGGCAACGTACCTAA | TCGTCGTCATCTTTCTGGTG | CAPs | *KpnI* |
| C5024^b^ | CATGAAGCGCCATAAGTTCA | GATTGGGCGTCCCTATTACC | CAPs | *DrdI* |
| K02497^c, d^ | TGGATGTCCGTTTGGAACTT | CCCAAGGGGTGCATGTAGT | CAPs | *TaqI* |
| K04079^c, d^ | CTTGCTATGGCGATCTTGTG | AAATCTTGAGCTTTGCCAGC | CAPs | *NsciI* |
| K05011^c^ | GAGAAGGGGTAGGGATTGGA | TGACAGACGCAAGAAGGATG | CAPs | *NheI* |
| K04482^c^ | TCGTGTGAAGGACGAAAACA | AAGGGACAGGGAAGAAGCAT | Sequencing | *–* |
| K00924^c^ | CCCTATGGCAGTGGTAGTTCA | TCAGAGAGGTGCTTGCCTTT | CAPs | *MnlI* |
| K02798^c^ | AGAGCTAGGACAATGCAAAGG | ACCAGCAAAGTAGGCATCAG | Sequencing | *–* |
| K04626^c^ | ACCAACTTCTAACGCCCAAG | CAGGCATACCCTCAATAGCA | Sequencing | *–* |
| K04569^c^ | TCAGTCAATGCTTGGGTCTG | GAAGTTGCGTCCCGTGATA | Sequencing | *–* |
| K00659^c, d^ | TGATTCCCCCATTTTCTTTG | GCACTTTCACCCCTACAACC | CAPs | *HinfI* |
| K02249^c^ | CACAAGATCATTTCGCATGG | GGCTAACCGTTTTCCCTCTC | CAPs | *BtgI* |

Supplemental Table S4. Primer sequences employed for qPCR, RACE PCR and RNA *in situ* hybridization experiments.

| Primer Name | Sequence |
| --- | --- |
| Absolute quantification PCR | |
| Forward: | 5′-GGATGGGTTGATGGTGGATT-3′ |
| Reverse: | 5′-AATGGACCCTTGCAGTTCAG-3′ |
| 5’ RACE-PCR |  |
| Primary Reverse: | 5′-GGAAGGGTGTGGAGCAATCGAAT-3′ |
| Nested Reverse: | 5′-GCTGTACTGTACGATGGAGGAGC-3′ |
| 3’ RACE-PCR |  |
| Primary Forward: | 5′-GCGGCCAAAACGTCTACAG-3′ |
| Nested Forward: | 5′-CGATGCAGAAACGAGAGGA-3′ |
| In situ hybridization |  |
| Antisense Forward: | 5′-GAGGTGCTCAAGAAATCAACC-3′ |
| Antisense Reverse: | 5′-AATTAACCCTCACTAAAGGGCGACAAGGTCATACGGTGAG-3′ |
| Sense Forward:: | 5′-TAATACGACTCACTATAGGGGAGGTGCTCAAGAAATCAACC-3′ |
| Sense Reverse: | 5′-CGACAAGGTCATACGGTGAG-3′ |

RACE-PCR, rapid amplification of cDNA ends-PCR

Supplemental Table S5. Primer sequences employed for the re-sequencing of the two candidates for the gene underlying the *cer-zv* mutation*.*

| Primer Name | Sequence | NO. |
| --- | --- | --- |
| Primers used for amplification and sequencing | | |
| BC871355_F1524^a^ | 5’-CCACTACCACCACCACGACT-3’ | 1 |
| BC871355_F1559^a^ | 5’-GCGGCCAAAACGTCTACAG-3’ | 2 |
| BC871355_F1682^a^ | 5’-GGGCAGATCCAATCAACAAC-3’ | 3 |
| BC871355_R1867^a^ | 5’-CGGCAACAGGAGGGTAAAG-3’ | 4 |
| BC871355_F1889^a^ | 5’-GGCTCACCTCACATCACACT-3’ | 5 |
| BC871355_R1989^a^ | 5’-TTTGAACGGAGTTGCAGATG-3’ | 6 |
| BC871355_F2251^a^ | 5’-GACAACACCCAAAACAGTGC-3’ | 7 |
| BC871355_F2472^a^ | 5’-CGGATAGTTTTAAGGTAGGG-3’ | 8 |
| BC871355_F2715^a^ | 5’-TAGCCGTCAAATCCGACAA-3’ | 9 |
| BC871355_F3044^a^ | 5’-GCAGAAGCAGGATCATAGACC-3’ | 10 |
| BC871355_F3434^a^ | 5’-GATTGCTCCACACCCTTCC-3’ | 11 |
| BC871355_R3562^a^ | 5’-GGGCAGCTCAGAAAGATCAC-3’ | 12 |
| BC871355_F3709 ^a^ | 5’-CGATGCAGAAACGAGAGGA-3’ | 13 |
| BC871355_R3990 ^a^ | 5’-CGACAAGGTCATACGGTGAG-3’ | 14 |
| BC871355_F4003 ^a^ | 5’-AATCCACCATCAACCCATCC-3’ | 15 |
| BC871355_R4193 ^a^ | 5′-AATGGACCCTTGCAGTTCAG-3′ | 16 |
| BC871355_R4272 ^a^ | 5’-GAGGCCGGTATACATCCACA-3’ | 17 |
| BC871355_R4338 ^a^ | 5’-ATCTCAGCGGTGATCCAAAC-3’ | 18 |
| AK249052_F20^b^ | 5’-CAAAACCAGAGGGAGAGCAG-3’ | 19 |
| AK249052_F218^b^ | 5’-CAAAACCAGAGGGAGAGCAG-3’ | 20 |
| AK249052_F546^b^ | 5’-CGTCGTTAAGGCCAAGGAG-3’ | 21 |
| AK249052_R1218^b^ | 5’- CCCAAGCCGACCTATCAGT-3’ | 22 |
| AK249052_F1425^b^ | 5’-AGTCGAGAGAGGCGAAAAGA-3’ | 21 |
| AK249052_R1516^b^ | 5’-GTGGCTTCTCTGTTCCCAAA-3’ | 22 |
| AK249052_R1737^b^ | 5’-CGCCTTCTCTCCACCACTTA-3’ | 23 |
| AK249052_R2010^b^ | 5’-TGCAAGTTCCCTTTCTCTGG-3’ | 24 |
| AK249052_R2308^b^ | 5’-TTGGCCGTTGATGATGTATGC-3’ | 25 |
